# Estimation of measurement error in plasma HIV-1 RNA assays near their limit of quantification

**DOI:** 10.1371/journal.pone.0171155

**Published:** 2017-02-02

**Authors:** Viviane D. Lima, Lu Wang, Chanson Brumme, Lang Wu, Julio S. G. Montaner, P. Richard Harrigan

**Affiliations:** 1 British Columbia Centre for Excellence in HIV/AIDS, Vancouver, British Columbia, Canada; 2 Division of AIDS, Department of Medicine, Faculty of Medicine, University of British Columbia, Vancouver, British Columbia, Canada; 3 Statistics Department, University of British Columbia, Vancouver, British Columbia, Canada; Instituto de Salud Carlos III, SPAIN

## Abstract

**Background:**

Plasma HIV-1 RNA levels (pVLs), routinely used for clinical management, are influenced by measurement error (ME) due to physiologic and assay variation.

**Objective:**

To assess the ME of the COBAS HIV-1 Ampliprep AMPLICOR MONITOR ultrasensitive assay version 1.5 and the COBAS Ampliprep Taqman HIV-1 assay versions 1.0 and 2.0 close to their lower limit of detection. Secondly to examine whether there was any evidence that pVL measurements closest to the lower limit of quantification, where clinical decisions are made, were susceptible to a higher degree of random noise than the remaining range.

**Methods:**

We analysed longitudinal pVL of treatment-naïve patients from British Columbia, Canada, during their first six months on treatment, for time periods when each assay was uniquely available: Period 1 (Amplicor): 08/03/2000–01/02/2008; Period 2 (Taqman v1.0): 07/01/2010–07/03/2012; Period 3 (Taqman v2.0): 08/03/2012–30/06/2014. ME was estimated via generalized additive mixed effects models, adjusting for several clinical and demographic variables and follow-up time.

**Results:**

The ME associated with each assay was approximately 0.5 log_10_ copies/mL. The number of pVL measurements, at a given pVL value, was not randomly distributed; values ≤250 copies/mL were strongly systematically overrepresented in all assays, with the prevalence decreasing monotonically as the pVL increased. Model residuals for pVL ≤250 copies/mL were approximately three times higher than that for the higher range, and pVL measurements in this range could not be modelled effectively due to considerable random noise of the data.

**Conclusions:**

Although the ME was stable across assays, there is substantial increase in random noise in measuring pVL close to the lower level of detection. These findings have important clinical significance, especially in the range where key clinical decisions are made. Thus, pVL values ≤250 copies/mL should not be taken as the “truth” and repeat pVL measurement is encouraged to confirm viral suppression.

## Background

Long-term suppression of plasma HIV-1 RNA levels (pVLs) below the quantification limit of clinically available assays is the critical goal for patients starting combination antiretroviral therapy (cART) [1]. Maintaining pVLs below this threshold has been shown to promote immune restoration, decrease morbidity and mortality associated with HIV disease, and prevent ongoing viral evolution and HIV transmission [1]. In most resource-rich settings, patients’ pVLs are monitored every 3 to 4 months for early diagnostic of treatment failure, and if failure is confirmed, treatment switch is often recommended. Frequency of monitoring varies in resource-limited settings depending on the availability of the test, however, this issue is rapidly evolving as a result of new guidelines and emerging technologies [2, 3].

Around the world, the Roche COBAS HIV-1 Ampliprep Amplicor Monitor ultrasensitive assay version 1.5 (or Amplicor v1.5) was used as the gold standard to measure pVLs for almost a decade (from 1997 to 2008). Its lower limit of quantification (i.e., 50 copies/mL) was adopted as the threshold defining effective cART [4]. In recent years, this assay was replaced by technically-simpler assays with a wider dynamic range [5]. Currently, the two most used assays are the Roche COBAS Ampliprep Taqman HIV-1 assay version 2.0 (or Taqman v2.0) or the Abbott Real*Time* HIV-1 RT-PCR assay. Even though pVLs based on these assays are routinely used to inform clinical management, it is important to stress that these measurements are not precise, and they are influenced by measurement error (ME) due to physiologic and assay variation [6, 7].

## Objective

To assess the ME of the Amplicor v1.5 and the Taqman v1.0 and v2.0 assays. Additionally, we examined whether there was any evidence that pVL measurements closest to the lower limit of quantification, where clinical decisions are made, were susceptible to a higher degree of random noise than the remaining range.

## Materials and Methods

### Data

Data were extracted from the British Columbia (BC) Centre for Excellence in HIV/AIDS in Vancouver, Canada. cART is distributed, free-of-charge, to all individuals living with HIV-1 according to specific guidelines consistent with those put forward by the International Antiviral Society-USA since 1996 [1, 8, 9].

Eligible patients were cART naïve, ≥ 19 years old, enrolled between January 1, 2000 and June 30, 2013 and followed until June 30, 2014. Initial cART regimens consisted of two nucleoside reverse transcriptase inhibitors as backbone, plus either a non-nucleoside reverse transcriptase inhibitor (NNRTI), a ritonavir-boosted protease inhibitor (bPI), an integrase inhibitor (IIN) or a CCR5 entry inhibitor (EI). Eligible individuals were also required to have a CD4 count and a pVL measured within six months of initiating cART.

CD4 cell counts were measured by flow cytometry, followed by fluorescent monoclonal antibody analysis (Beckman Coulter, Inc., Mississauga, Ontario, Canada). CD4 data was obtained from different laboratories across BC, covering >85% of all tests done in the province. All pVL measurements were done at the St Paul’s Hospital virology laboratory. Because of the systematic differences in measurement in the low pVL range, BC’s HIV treatment guidelines now use the threshold ≤250 copies/mL by Taqman v2.0 to define virologic suppression [10, 11]. Thus, all our analyses paid special attention to the data below this cut-off. In BC, approximately 90% of patients have clade B subtype, and only a small number have other subtypes (mostly clades A and C subtypes).

### Analysis

The first analysis consisted of examining all pVL measurements for each patient during their follow-up to detect any pattern in pVL results from each assay. Second, for estimating the ME of these assays, we restricted the data to the first six months of follow-up, since thereafter, the majority of our patients would have achieved viral suppression, and thus measuring the ME would be difficult since we do not have the exact pVL value below the assay’s limit of quantification. The third analysis consisted in examining the distribution of pVL measurements, between the lower limit of quantification of these assays and 1000 copies/mL, to assess whether there was evidence that some low level pVL measurements may simply be assay "false positive" values, rather than resulting from other factors such as intermittent periods of treatment non-adherence. Note that this is the same range used to define a viral load “blip” [12, 13].

Based on these assays, we stratified the data and analysis into three mutually exclusive periods in which only one assay was used: Period 1 (Amplicor; range of quantification 50–100,000 copies/mL): March 8, 2000 to February 1, 2008; Period 2 (Taqman v1.0; 40–1,000,000 copies/mL): January 7, 2010 to March 6, 2012; Period 3 (Taqman v2.0; 40–1,000,000 copies/mL): March 7, 2012 to June 30, 2014.

The outcome in this study was log_10_-transformed pVL measured longitudinally from start of cART up to six months. Explanatory variables, measured at baseline, included: age (continuous), gender (male or female), history of injection drug use (yes, no or unknown), CD4 cell count, adherence level measured between baseline and 12 months since cART initiation (<40%, 40% to <80%, 80% to <95% or ≥95%), and regimen (NNRTI, bPI or IIN/EI). We used the adherence measured at 12 months since it is a more reliable measure than that measured at six months due to different prescription refill patterns across patients. Adherence level was estimated by dividing the number of months of medications dispensed by the number of months of follow-up. In different studies, this adherence measure was associated with virologic outcomes in the short and long terms [14, 15]. We also included in the model the follow-up (in months) from baseline to the date in which each pVL was measured.

To estimate the ME inherent in each assay, we used generalized additive models with random effects [16–18], assuming a first-order autoregressive correlation structure. The advantage of using these models rely on the fact that they: (1) provide flexibility in modeling non-linear trends in pVL measurements; (2) adjust for both the inter- and intra-patient variation that, otherwise, can bias our results; (3) are flexible to accommodate unbalanced data (i.e., different number of pVL measurements per patient); and (4) control for correlated pVL data collected for each patient over time. This methodology has been previously used to estimate the ME in CD4 cell count measurements and can be extended to estimating the ME in any PCR (Polymerase Chain Reaction)-based assays. We run these models in R© version 3.2.2. Multivariable explanatory models were built using a modified backward stepwise technique based on the Akaike Information Criterion and Type III p-values [19]. We used a cubic regression spline to smooth the non-linear time trend. Goodness-of-fit assessment was based on the adjusted R^2^, the percentage of the deviance explained, and a test to check the appropriateness of the number of knots in the model [16, 17].

Thus, for each period, we fitted the following model:

$$log_{10}\;\left(Plasma\;HIV\text{-}1\;RNA\;Level_{ij}\right)=b_{0i}+b_{1i}\;Time_{ij}+\beta_{0}+s\left(Time_{ij}\right)+\beta_{1}\;Age_{i}+\beta_{2}\;Gender_{i}+\beta_{3}\;History\;of\;Injection\;Drug\;Use_{i}+\beta_{4}\;CD4_{i}+\beta_{5}\;Adherence_{i}+\beta_{6}\;Regimen_{i}+\varepsilon_{ij}$$

where *β*_0_, …, *β*_*6*_ represent fixed effect parameters; $b_{0i}∼N(0,\sigma_{0i}^{2})$ *and* $b_{1i}∼N(0,\sigma_{1i}^{2})$ are normally distributed random effect parameters with mean zero and variance $\sigma_{0i}^{2}$ and $\sigma_{1i}^{2}$, respectively; *s*( ) denotes the cubic regression spline function; and *ε*_*ij*_ *~ N(0*,*σ*^*2*^*)* are the residuals which is assumed to be normally distributed with mean zero and variance; for *i* = 1,…,*N* (i.e., the number of patients), *j* = 1,…,*T*_*i*_ (i.e., the number of pVLs per patient). Note that in this case, the random effect terms were responsible for modeling the inter- and intra-patient variation, the cubic regression spline function were responsible for modeling the non-linear pVL trends, and the ME were estimated by taking the square root of the estimated variance of the residuals (i.e., *σ*). Note that the residuals are calculated by taking the difference between the observed and fitted pVL values, and they are the random noise in the model.

## Results

**Fig 1:**
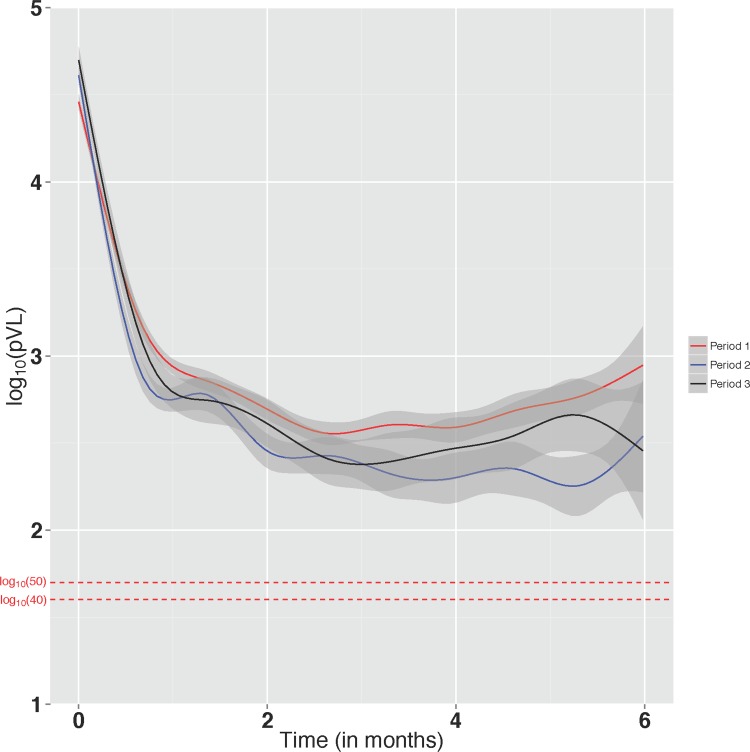
Trajectory in the mean log_10_ transformed plasma HIV-1 RNA levels (pVL), and associated 95% confidence interval for the mean (grey area around each trajectory), from antiretroviral treatment initiation to six months of follow-up. Period 1 (Amplicor): March 8, 2000 to February 1, 2008; Period 2 (Taqman v1.0): January 7, 2010 to March 6, 2012; Period 3 (Taqman v2.0): March 7, 2012 to June 30, 2014.

We longitudinally followed 1933 patients in Period 1, 979 in Period 2, and 429 in Period 3. Overall, in all periods of observation, patients were more likely to be male, to have no history of injection drug use, to have started treatment on a bPI-based regimen, and to have adherence ≥95% during the first year on therapy (Table 1). In terms of CD4 cell count at cART initiation, in Period 1, 60% of patients had CD4 cell count <200 cells/mm^3^, while in the other periods, the distribution of baseline CD4 cell count was quite similar across all categories. Additionally, in all periods, at the start of cART, the median age was just over 40 years, median pVL was approximately 5.0 log_10_ copies/mL, and the median number of pVL measurements per patient ranged from 2 to 3 (Table 1). The trajectories of the mean pVL (and associated 95% confidence interval) for these periods from start of cART up to six months were very similar and they are presented in Fig 1.

**Table 1:** Patient demographic and clinical characteristics by period of plasma HIV-1 RNA level measurements. Period 1 (Amplicor): March 8, 2000 to February 1, 2008; Period 2 (Taqman v1.0): January 7, 2010 to March 6, 2012; Period 3 (Taqman v2.0): March 7, 2012 to June 30, 2014.

Variable	Period 1	Period 2	Period 3
	N = 1933	N = 979	N = 429
**Gender, n(%)**			
Male	1584 (82%)	793 (81%)	334 (78%)
Female	349 (18%)	186 (19%)	95 (22%)
**History of Injection Drug Use, n(%)**			
No	869 (45%)	451 (46%)	181 (42%)
Yes	762 (39%)	292 (30%)	91 (21%)
Unknown	302 (16%)	236 (24%)	157 (37%)
**Baseline CD4 (cells/mm**^**3**^**), n(%)**			
<200	1157 (60%)	249 (25%)	99 (23%)
200 to 349	589 (30%)	285 (29%)	94 (22%)
350 to 499	130 (7%)	241 (25%)	110 (26%)
≥500	57 (3%)	204 (21%)	126 (29%)
**Adherence during first year of follow-up, n(%)**			
<40%	140 (7%)	42 (4%)	16 (4%)
40% to <80%	246 (13%)	124 (13%)	53 (12%)
80% to <95%	240 (12%)	156 (16%)	66 (15%)
≥95%	1307 (68%)	657 (67%)	294 (69%)
**First antiretroviral regimen, n(%)**			
NNRTI	852 (44%)	490 (50%)	183 (43%)
bPI	1081 (56%)	468 (48%)	214 (50%)
IIN/EI	0 (0%)	21 (2%)	32 (7%)
**Age (years)**			
Median	42	41	41
25^th^–75^th^ percentile	36–49	33–48	32–49
**Baseline plasma HIV-1 RNA level (log**_**10**_ **copies/mL)**			
Median	5.0	4.6	4.8
25^th^–75^th^ percentile	4.6–5.0	4.1–5.2	4.3–5.2
**Number of plasma HIV-1 RNA level measurements per patient**			
Median	2	3	3
25^th^–75^th^ percentile	1–3	2–4	2–4
Minimum–Maximum	1–10	1–8	1–10

**Footnote:** NNRTI: non-nucleoside reverse transcriptase inhibitor; bPI: ritonavir (dose of <400mg/day) boosted protease inhibitor; IIN: integrase inhibitor; EI: CCR5 entry inhibitor.

Based on the multivariable model, the estimated ME for all periods was fairly similar ranging from 0.52 to 0.55 log_10_ copies/mL (Table 2). Secondly, we examined the frequency of all pVLs, across all patients, between 50–1000 copies/mL for Period 1 and between 40–1000 copies/mL for Periods 2 and 3. We noted that pVL measurements closest to the lower limit of quantification of each of the assays were strongly systematically overrepresented (i.e., not random), with the prevalence decreasing monotonically as the reported pVL value increased. To illustrate this point, we calculated summary statistics for each reported pVL value in the strata 50–99 or 40–99, 100–249, 250–499 and 500–1000 copies/mL (Table 3). For example, in Period 1, we observed that the median number of repeated observations per pVL value in each of these strata was, respectively, 24 (Q1-Q3: 19–32), 9 (Q1-Q3: 7–12), 4 (Q1-Q3: 2–5) and 2 (Q1-Q3: 1–3). Looking at the non-stratified pVL data we observed that there were 33 observations of “51 copies/mL”, 50 observations of “52 copies/mL”, 49 observations of “53 copies/mL”, 31 observations of “54 copies/mL”, while approximately 87% of individual pVLs between 500–1000 copies/mL had 0, 1, 2 or 3 observations.

**Table 2:** Estimated measurement error from the multivariable generalized additive mixed effects models. Period 1 (Amplicor): 08/03/2000-01/02/2008; Period 2 (Taqman v1.0): 07/01/2010-07/03/2012; Period 3 (Taqman v2.0): 08/03/2012-30/06/2014.

Time Periods	Measurement Error (95% Confidence Interval)	Goodness-of-fit Statistics	
		Adjusted R^2^	Percent of Deviance Explained
Period 1	0.55 (0.53–0.57) log_10_ copies/mL	0.70	78%
Period 2	0.53 (0.51–0.55) log_10_ copies/mL	0.83	88%
Period 3	0.52 (0.49–00.55) log_10_ copies/mL	0.84	89%

**Table 3:** Number of observations per plasma HIV-1 RNA levels (pVL) between the lower limit of quantification of each assay and 1000 copies/mL. Period 1 (Amplicor): 08/03/2000-01/02/2008; Period 2 (Taqman v1.0): 07/01/2010-07/03/2012; Period 3 (Taqman v2.0): 08/03/2012-30/06/2014.

Plasma HIV-1 RNA levels (copies/mL)	Number of Observations per Value in Each Strata				
	Minimum	Median	25^th^ Percentile	75^th^ Percentile	Maximum
**Period 1**					
50–99	11.0	24.0	19.0	32.0	50.0
100–249	2.0	9.0	7.0	12.0	23.0
250–499	1.0	4.0	2.0	5.0	11.0
500–1000	1.0	2.0	1.0	3.0	9.0
**Period 2**					
40–99	3.0	11.0	8.0	15.0	22.0
100–249	1.0	4.0	2.0	5.0	11.0
250–499	1.0	2.0	1.0	2.0	10.0
500–1000	1.0	1.0	1.0	2.0	4.0
**Period 3**					
40–99	1.0	5.5	4.0	8.3	16.0
100–249	1.0	2.0	1.0	3.0	9.0
250–499	1.0	1.0	1.0	1.3	4.0
500–1000	1.0	1.0	1.0	1.0	3.0

In the last analysis, we examined the model residuals to assess whether the pVL measurements may have been susceptible to different degrees of random noise along the range of quantification of the assays (Table 4). Based on the residual analysis, to address our second objective, we stratified the model residuals using the cut-off 250 copies/mL. In Period 1, for example, we observed that the median model residuals for pVL ≤250 copies/mL was -0.313 (Q1-Q3: -0.514; -0.184), and for pVL >250 copies/mL the median was 0.142 (Q1-Q3: -0.113; 0.390), which in absolute terms, this last value was 2.2 times lower than that for the lower range. Note that for the other periods, the model residuals in comparing these two pVL strata were slightly more distinct, being 3.5 and 2.4 times in Periods 2 and 3, respectively.

**Table 4:** Distribution of residuals from the generalized additive mixed effects models. Period 1 (Amplicor): 08/03/2000-01/02/2008; Period 2 (Taqman v1.0): 07/01/2010-07/03/2012; Period 3 (Taqman v2.0): 08/03/2012-30/06/2014.

Plasma HIV-1 RNA level category	Difference between observed and fitted plasma HIV-1 RNA level valueMedian (25th-75th percentile)		
	Period 1	Period 2	Period 3
≤ 250 copies/ml	-0.313 (-0.514; -0.184)	-0.124 (-0.277; -0.003)	-0.164 (-0.322; -0.027)
> 250 copies/ml	0.142 (-0.113; 0.390)	0.035 (-0.198; 0.284)	0.068 (-0.135; 0.269)

## Discussion

Based on this study, the estimated ME associated with each assay was approximately 0.50 log_10_ copies/mL, which is consistent the literature [20]. Thus, patients and physicians should be aware that a pVL of 50 copies/mL really means that it is likely that the “true” pVL is between 16 and 158 copies/mL, or a pVL of 250 copies/mL really means that it is likely that the “true” pVL is between 79 and 791 copies/mL. We also observed that there is substantial increase in random noise in measuring pVLs <250 copies/mL, especially close to the lower level of detection of each assay. Interestingly, we also detected a pattern such that pVLs near each assay’s lower limit of quantification were over-represented than values ≥500 copies/mL. There are different possible explanations for this finding. First, each of these repeated measurements may be a genuine representation of underlying distribution of pVLs. Second, repeated pVL testing improves the likelihood of testing below the limit of quantification. Thus, due to ME, a patient whose “true” pVL remains above the limit of quantification can test below this cut-off by chance. Consequently, the more tests are performed in a given patient with a low detectable pVL, the higher the likelihood that at least one measurement will be below the limit of quantification. Finally, low detectable values near the limit of quantification could represent assay’s “false positive” results [12, 13]. As important clinical decisions (e.g., change in drug regimen due to virologic failure) are made based on this range, this study highlights the fact that single pVL results are not reliable given the ME and random noise pertaining to currently used assays, and the likelihood of a pVL being equal to 51 copies/mL, for example, is very small. Instead, physicians should rely on confirmatory retesting to ascertain pVL-based outcomes in patients.

There are some features of this study worth mentioning. Our cohort is unique in that it was built within a population-based program where all patients had access to the same free cART options, medical care and laboratory monitoring, with no co-payments or deductibles. This minimizes treatment access as a possible confounding factor. Second, our database is comprehensive as it captures 100% of cART refills and pVL measurements, and approximately 85% of CD4 cell counts done in BC. Third, this study was based on cART-naïve patients, thus making our results not influenced by confounding by previous therapy use. Fourth, we relied on covariates measured at baseline to estimate the ME. As shown, all models had at ≥78% of their deviance explained, which is considerable, however it also suggests that there is room for improvement by means of considering additional covariates (if available). One of these covariates could be CD4 cell count measured longitudinally. However, since CD4 cell counts are also susceptible to ME, more complex models can be used to assess whether model fit can be improved. Additionally, since our analyses were restricted to pVL measured up to the sixth month of follow-up, we can extend this analysis to adjust for left-censored data. However, these models are highly complex and currently available statistical software is not capable of handling these models. We also should acknowledge that some of these pVL assays may be susceptible to artefacts such as viral diversity, virus subtype, and primers used in primary plasma preparation tubes, which may contribute to the measurement variability here described [21–26]. Although these are legitimate concerns, it should be noted that the virology laboratory does not use these tubes, the same primers were used in all measurements, and >90% of our patients have clade B subtype. Some may argue whether pVLs of patients during acute HIV infection could have contributed to the over-representation of viral loads close to the limits of quantification. To address this concern, given that in our database there is no precise date in which the patient tested positive for HIV, the first pVL date is the best proxy we have. Thus, we decided to examine the distribution of the first pVL of patients (before start of ART), overall and for those with CD4 >500 and >750 cells/mm^3^ at the time in which the first pVL was obtained. We did this analysis for all three periods. We observed that the first pVL was high for the overall cohort (≥3.81 log_10_ copies/mL) although slightly lower than the pVL at the start of ART. Also at the time of first pVL, only a small number of patients had CD4>750 cells/mm^3^ (i.e., indicative of recent infection) and their viral load was ≥2.78 log_10_ copies/mL. Thus, we do not think that there was an over-representation of pVLs of patients during acute infection. Finally, only by looking at thousands of pVL measurements, we were able to observe the high frequency of pVLs values below 250 copies/mL, especially close to the lower limit of quantification of these assays. To our knowledge, this is the first study able to identify this phenomenon.

In conclusion, our results demonstrate that although the ME was stable across assays, there is substantial increase in random noise as the pVL approaches the assays’ lower level of detection. These findings have important clinical significance, as they validate the use of the 250 copies/mL cut-off to define virologic suppression, and reinforce the fact that confirmatory pVL measurements should be used to inform clinical decisions, especially when the pVL is close to the lower limit of quantification of the assay.

## Ethical Approval

The British Columbia Centre for Excellence in HIV/AIDS received approval for this study from the University of British Columbia ethics review committee at the St Paul’s Hospital, Providence Health Care site (H05-50123). The study complies with the BC’s Freedom of Information and Protection of Privacy Act. The study was conducted primarily using anonymized administrative databases, and therefore specific informed consent was not required.

## Abbreviations

HIV-1: Human immunodeficiency virus of type1
pVL: plasma HIV-1 RNA level
copies/mL: copies per milliliter
cells/mm^3^: cells per cubic millimetre
cART: combination antiretroviral therapy
ME: measurement error
BC: British Columbia
NNRTI: non-nucleoside reverse transcriptase inhibitor
bPI: ritonavir-boosted protease inhibitor
IIN: integrase inhibitor
EI: CCR5 entry inhibitor
CCR5: C-C chemokine receptor type 5
AIC: Akaike Information Criterion
Q1: 25^th^ percentile
Q3: 75^th^ percentile
95% CI: 95% confidence interval
